# PO_2_ Cycling Reduces Diaphragm Fatigue by Attenuating ROS Formation

**DOI:** 10.1371/journal.pone.0109884

**Published:** 2014-10-09

**Authors:** Li Zuo, Philip T. Diaz, Michael T. Chien, William J. Roberts, Juliana Kishek, Thomas M. Best, Peter D. Wagner

**Affiliations:** 1 Radiologic Sciences and Respiratory Therapy Division, School of Health and Rehabilitation Sciences, Davis Heart and Lung Research Institute, The Ohio State University College of Medicine, Columbus, Ohio, United States of America; 2 Department of Medicine, University of California San Diego, La Jolla, California, United States of America; 3 Department of Biological Sciences, Oakland University, Rochester, Michigan, United States of America; 4 Division of Sports Medicine, Department of Family Medicine, Sports Health and Performance Institute, The Ohio State University, Columbus, Ohio, United States of America; 5 Department of Biology, Kalamazoo College, Kalamazoo, Michigan, United States of America; 6 Division of Pulmonary, Allergy, Critical Care & Sleep Medicine, The Ohio State University Wexner Medical Center, Columbus, Ohio, United States of America; Chinese Academy of Sciences, China

## Abstract

Prolonged muscle exposure to low PO_2_ conditions may cause oxidative stress resulting in severe muscular injuries. We hypothesize that PO_2_ cycling preconditioning, which involves brief cycles of diaphragmatic muscle exposure to a low oxygen level (40 Torr) followed by a high oxygen level (550 Torr), can reduce intracellular reactive oxygen species (ROS) as well as attenuate muscle fatigue in mouse diaphragm under low PO_2_. Accordingly, dihydrofluorescein (a fluorescent probe) was used to monitor muscular ROS production in real time with confocal microscopy during a lower PO_2_ condition. In the control group with no PO_2_ cycling, intracellular ROS formation did not appear during the first 15 min of the low PO_2_ period. However, after 20 min of low PO_2_, ROS levels increased significantly by ∼30% compared to baseline, and this increase continued until the end of the 30 min low PO_2_ condition. Conversely, muscles treated with PO_2_ cycling showed a complete absence of enhanced fluorescence emission throughout the entire low PO_2_ period. Furthermore, PO_2_ cycling-treated diaphragm exhibited increased fatigue resistance during prolonged low PO_2_ period compared to control. Thus, our data suggest that PO_2_ cycling mitigates diaphragm fatigue during prolonged low PO_2_. Although the exact mechanism for this protection remains to be elucidated, it is likely that through limiting excessive ROS levels, PO_2_ cycling initiates ROS-related antioxidant defenses.

## Introduction

Low oxygen/hypoxic conditions can significantly reduce skeletal muscle contraction [1]. In normal resting muscle, it has been reported that skeletal muscles, such as the diaphragm, produce reactive oxygen species (ROS) including hydrogen peroxide (H_2_O_2_) [2]. However, when the diaphragm is repetitively stimulated, these muscle fibers generate excessive ROS leading to oxidative stress with accelerated fatigue development [2]. Moreover, the production of ATP is driven by electron transmission through mitochondrial complex I to complex IV, creating a proton gradient across the inner mitochondrial membrane (IMM) and triggering ATP synthesis [3], [4]. Through this mechanism, a small portion of electrons may leak out of the IMM and react with adjacent oxygen molecules to produce superoxide anions, H_2_O_2_, and other ROS. Under prolonged low PO_2_ conditions, the physiological concentration of O_2_ is altered which results in increased uncoupling between O_2_ and electron flow, ultimately causing ROS overproduction [5].

A variety of cellular preconditioning pathways associated with muscular protection have been proposed. For instance, ischemic preconditioning (IPC), which consists of ischemic-reperfusion cycles produced by variations in low-high PO_2_ levels, has been used to prevent cardiac muscle injuries [6]. In addition, IPC initiates intracellular protein kinase pathways, resulting in increased activation of antioxidant enzymes such as catalase [6]. IPC also plays a critical role in protecting the heart against ischemia-reperfusion injuries by opening mitochondrial ATP sensitive potassium channels (mK_ATP_) [7]. The mK_ATP_ channels are regulated by several factors, including adenosine, H^+^, and/or protein kinase C. Thus, these mediators may partially contribute to the protective response involved in preconditioning therapies [8], [9]. Similar to IPC, PO_2_ cycling preconditioning, which consists of brief periods of lower-higher PO_2_, significantly protects heart muscle cells subjected to prolonged ischemia by decreasing ROS-induced cell death [7], [10]. In addition, human studies have shown that intermittent low oxygen exposure at low altitude significantly increases an aircraft crew's adaptation to low oxygen conditions experienced at high altitude [11]. Since the method of both IPC and PO_2_ cycling preconditioning involves brief periods of low and high oxygen levels, it is possible that PO_2_ cycling follows a similar molecular pathway as IPC. Furthermore, it has been shown that a protocol consisting of PO_2_ cycling provides a protective response against mesenchymal stem cell (MSC) apoptosis through phosphorylation of extracellular regulated kinase (ERK1/2) and protein kinase B (AKT) [12]. Therefore, it is possible that these signaling factors also may be involved in the molecular mechanism of PO_2_ cycling in skeletal muscle.

Moreover, lower PO_2_ or hypoxic conditions may cause changes in the cytosolic redox equilibrium, resulting in a rise in NADPH. The increase subsequently stimulates inositol triphosphate (IP3) receptor mediated release of Ca^2+^ from the endoplasmic reticulum. This release of Ca^2+^ activates important cell survival signaling pathways, which may potentially contribute to the preconditioning response during lower PO_2_ stress [13]. However, the redox mechanism of PO_2_ cycling preconditioning particularly in respiratory skeletal muscle has not been fully elucidated. The ultimate importance of the work is to develop treatments for those who may experience respiratory muscle fatigue. It is likely that PO_2_ cycling initiates ROS-related protective responses, particularly in a key muscle of respiration such as the diaphragm, which must be active throughout life [14].

In this study, we tested the hypothesis that PO_2_ cycling preconditioning decreases intramuscular ROS levels and enhances diaphragm muscle function. Our results demonstrate that PO_2_ cycling effectively reduces diaphragm fatigue during a prolonged low PO_2_ (40 Torr) condition, which is accompanied by decreased intracellular ROS levels. These findings provide insight into the molecular redox mechanism of PO_2_ cycling in diaphragmatic skeletal muscle exposed to a lower PO_2_ environment.

## Materials and Methods

### Animals

Male adult C57BL/6 mice (∼20–30 g, average age of ∼3 mo.) were used in accordance with the Ohio State University's and Oakland University's Institutional Laboratory Animal Care and Use Committee (IACUC). We strictly followed the Guide for the Care and Use of Laboratory Animals of the National Institutes of Health and Ethics of Animal Experiments. Mice were anesthetized via intraperitoneal (IP) injection with a combination of ketamine (70 mg/kg) and xylazine (10 mg/kg). The diaphragm was quickly removed from the mouse and muscle strips (∼0.5 cm wide, ∼1 cm long, 1–2 strips obtained from each mouse) were dissected from the diaphragm with the corresponding rib attachment and central tendon. After isolation, the muscle strip was kept in Ringer's solution (in mM: 21 NaHCO_3_, 1.0 MgCl_2_, 1.2 Na_2_HPO_4_, 0.9 Na_2_SO_4_, 2.0 CaCl_2_, 5.9 KCl, 121 NaCl, and 11.5 glucose), at 37°C.

### PO_2_ cycling and muscle function measurement

Function experiments were performed in a contraction chamber (model 800 MS; Danish Myo Technology, Denmark), with the central tendon of the muscle strip sutured to a mobile lever, which was used to adjust the muscle length for optimal performance. The opposite end of the strip was secured to a force transducer (detection range 0–1,600 mN). After being mounted, muscle optimal length (L_0_) was set as the baseline tension and no adjustments were made throughout the muscle function experiments. All muscles were electrically stimulated (S48 stimulator; Grass Technologies, RI) using square-wave pulses (250-ms train duration, 0.5-ms pulse duration, 70 Hz, 30 V), following previous skeletal muscle function protocols [15], [16]. The A-D board (model ML826; AD instruments, CO) converted the analog signals to digital data, and LabChart 7.3.1 software was used to analyze the function data. The muscle was equilibrated in Ringer's solution for 20–30 min. During the function experiments, the treated muscle strips were switched to a Ringer's solution equilibrated with PO_2_ of 40 Torr O_2_ (lower) for 2 min, followed by PO_2_ of 550 Torr O_2_ (higher) for 2 min. This PO_2_ cycle was repeated five times, followed by a prolonged 30-min 40 Torr PO_2_ period. During this exposure, muscle contractility was evaluated, in order to determine the effect of PO_2_ cycling on the muscle function. The chamber solution during lower PO_2_ was found to be 40 Torr and during higher PO_2_, 550 Torr. In the middle of the 40 Torr PO_2_ period (from 15–20 min), the muscle was stimulated for 5 min at 37°C and muscle tension development was recorded. The control group followed an identical protocol as the experimental group except for the one intervention of PO_2_ cycling. Following the removal of the attached rib bone and excess tendon, the diaphragm was first air dried which was followed by a 30-min oven drying. The dry mass was then determined using an analytical balance. To reduce random effects due to animal variance, all function data were normalized by dry weight of the muscle strip (mN/mg).

Regarding the H_2_O_2_ treatment with PO_2_ cycling group for low PO_2_ contraction measurements, the muscle strips were treated the same as above except that after PO_2_ cycling, we added H_2_O_2_ into the muscle contraction solution. Although H_2_O_2_ may degrade rapidly, at sufficient levels, it can enter the cell freely and affect the intracellular activity [17]. Specifically, the muscle was loaded with Ringer's solution with adequate H_2_O_2_ (50 µM) for 15 min prior to the 5-min contractions in low PO_2_ conditions. In addition, the time to reach 50% (T_50_) of the initial tension in contracting diaphragm muscle during a 5-min low PO_2_ contraction period was recorded in control, PO_2_ cycling, and PO_2_ cycling + H_2_O_2_ groups.

The effect of varying H_2_O_2_ dosage on muscle tension development was evaluated. The muscle strips were prepared in the same manner as mentioned above. During a high PO_2_ (550 Torr) period, each muscle strip was equilibrated with Ringer's solution for 15 min followed by a 15 min incubation with a particular dosage of H_2_O_2_ (0 µM, 50 µM, 100 µM, 1 mM, to 10 mM, respectively). After incubation, each diaphragm strip was stimulated for 5 min at 37°C and the muscle tension development was recorded.

For the H_2_O_2_ treatment group with no PO_2_ cycling for high PO_2_ contraction measurements, the muscle strips were exposed to high PO_2_ (550 Torr). Each muscle strip had two independent 5-min contraction periods, which were separated by a 60-min rest period. The muscle was loaded with H_2_O_2_ (50 µM) for 15 min followed by the first 5-min contractile period. The H_2_O_2_ was then washed out with fresh Ringer's solution, and the muscle was kept for a 60-min rest period before a second 5-min contraction bout in the absence of H_2_O_2_. This protocol was also performed in a blocked order to ensure the statistical value.

### Confocal studies

To analyze the effects of PO_2_ cycling treatment on ROS levels in superfused diaphragm, confocal microscopy was used to measure real-time ROS (H_2_O_2_) production in both PO_2_ cycling-treated and control diaphragm tissue. Specifically, each muscle strip was loaded with a 40 µM solution of dihydrofluorescein diacetate (Hfluor-DA; stock in dimethyl sulfoxide; Sigma-Aldrich) for 30 min. The dye diffused into the intramuscular compartment and was able to chemically react with intracellular ROS (mainly H_2_O_2_) resulting in enhanced florescence. For statistical purposes, one muscle strip was taken from each mouse. We used five mice for control and five mice for PO_2_ cycling treatment. A laser scan confocal microscopy system (Nikon confocal microscope D-Eclipse C1 system) recorded fluorescent emission signals from the tissue sample in a glass bottom culture dish (MatTek Corporation, Ashland, MA) in real time. The treated muscle strips were mounted and superfused with Ringer's solution, followed by PO_2_ cycling treatment, which was a similar method as described above for the muscle function experiments. To ensure an accurate oxygen level, the chamber was sealed except for the tubing inlets containing gas bubbling and superfusate as well as the temperature probe. The superfusate solution was fully saturated with designated gas and preheated to ensure the temperature in the chamber remained at 37°C. The strips were then subjected to a 10-min baseline period (PO_2_ of 550 Torr) and a subsequent 30-min 40 Torr PO_2_ period at 37°C. The control muscles followed the same protocol except that there was no PO_2_ cycling treatment. During the 40-min experimental period (10 min for baseline of 550 Torr PO_2_ and 30 min 40 Torr PO_2_ period), we captured an image (512×512 pixels) every 5 min and calculated the mean fluorescence to determine intramuscular ROS levels. To reduce the signal-to-noise ratio, each recorded image was an average from eight sequentially scanned images within 5 s at each time point. The setup parameters for the confocal imaging system were listed as follows: laser, argon; pinhole: medium or large; excitation, 488 nm; emission, 535±25 nm. The baseline autofluorescence was kept at a minimum and did not interfere with the ROS signal in our set-up. To reduce photobleaching or photodamaging, the laser power was set at ∼15% without noticeably sacrificing image quality. To reduce imaging saturation due to possible excessive ROS in 40 Torr PO_2_ conditions, the PMT gain was set as low as possible from the start of each experiment. In order to verify that the increased fluorescence signal was due to ROS, a series of antioxidant experiments were conducted. The glutathione peroxidase mimic, ebselen (30 µM), which is an effective ROS scavenger particularly for skeletal muscle tissue [5], was utilized. The animals were divided into 4 experimental groups, including control, PO_2_ cycling, ebselen, and PO_2_ cycling + H_2_O_2_. In each experimental group, five muscle samples were directly isolated from five fresh isolated diaphragms. For each experiment, we were able to measure ROS levels from ∼8–10 muscle fibers in each image field.

In preliminary studies, we found that the PO_2_ cycling protocol did not change the fluorescence baseline in the current set-up (data not shown). Each acquired image was analyzed with Adobe Photoshop element 6.0 and the final images were presented in a 300 DPI resolution with LZW compression.

### Statistics

By performing the power analysis on the sample, we defined the PO_2_ cycling effect on the skeletal muscle force development as well as intracellular ROS formation. For instance, we determined the PO_2_ cycling effect on multiple groups including control, antioxidant (ebselen) treatment and H_2_O_2_ application, using the prospective means across these groups. We derived the power when the sample size was ∼5 or greater mice per group by calculating the standard deviation. In addition, data were analyzed using a multi-way ANOVA with the animal as a variable, and expressed as means ± SE. The differences between the two treatments were statistically determined by a series of post-ANOVA contrast analyses using JMP software (SAS Institute, Cary, NC). Specifically, the post-ANOVA contrasts involve the comparison among all the groups of subjects and the display of the statistical difference between each pair of data. The treated groups, such as PO_2_ cycling, antioxidant (ebselen) treatment and H_2_O_2_ application, were used to compare with the control group, revealing any potential significance. *P*<0.05 was regarded to be significant.

## Results

**Figure 1:**
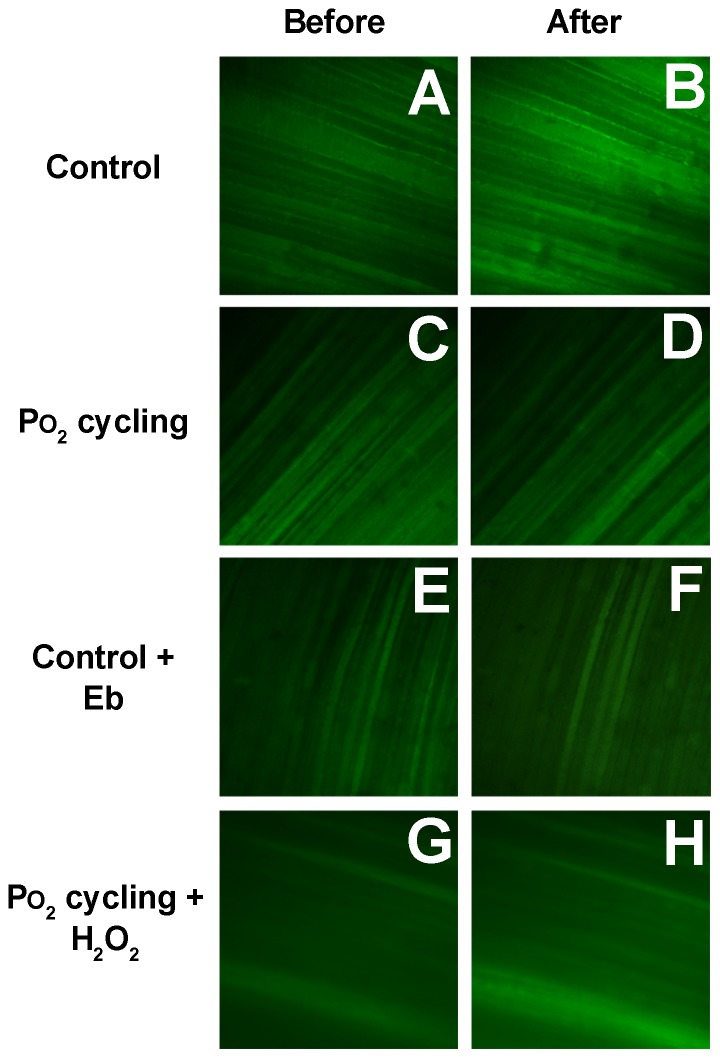
Representative ROS images from Hfluor-loaded diaphragm tissue. A: control muscle before 40 Torr PO_2_. B: the same area of A at the end of 40 Torr PO_2_. C: PO_2_ cycling treated muscle before 40 Torr PO_2_. D: the same area of C at the end of 40 Torr PO_2_. E: Ebselen (Eb) treated muscle before 40 Torr PO_2_. F: the same area of E at the end of 40 Torr PO_2_. G: PO_2_ cycling + H_2_O_2_ treated muscle before 40 Torr PO_2_. H: the same area of G at the end of 40 Torr PO_2_.

Representative confocal images of the same muscular area in each group are illustrated in Fig. 1. Fluorescence (green color), which represents ROS levels, increased substantially at the end of prolonged 40 Torr PO_2_ period (30 min, Fig. 1B) compared to baseline in the control group (Fig. 1A). However, after PO_2_ cycling treatment, fluorescence displayed no significant change at the end of 40 Torr PO_2_ (Fig. 1D) when compared to baseline (Fig. 1C). The disappearance of increased fluorescence emission in PO_2_ cycling treatment demonstrated that PO_2_ cycling effectively suppressed 40 Torr PO_2_-induced intracellular ROS levels in the diaphragm. In addition, ebselen treated muscle strips showed no fluorescence increase at the end of 40 Torr PO_2_ exposure (Fig. 1F). Interestingly, exogenous addition of H_2_O_2_ (50 µM) mitigated the antioxidant effect of PO_2_ cycling (Fig. 1H).

**Figure 2:**
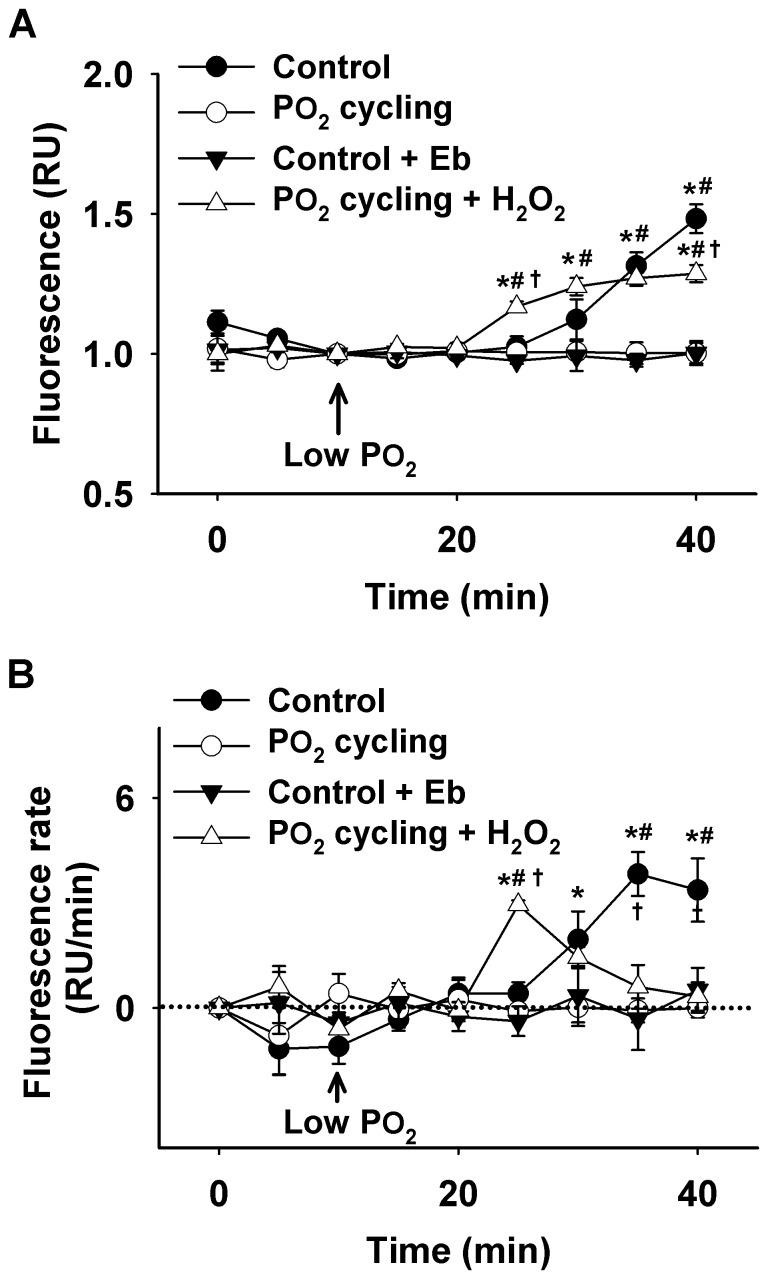
Intracellular ROS fluorescence and fluorescence rate under 40 Torr PO_2_. A: averaged fluorescence data recorded in a relative unit (RU). Data showed intracellular ROS levels from control, PO_2_ cycling, ebselen (Eb), and PO_2_ cycling + H_2_O_2_ treated diaphragm muscle (*significantly different from PO_2_ cycling, *P*<0.05; ^#^significantly different from Eb treatment, *P*<0.05; ^†^significantly different from control, *P*<0.05). B: intracellular ROS burst represented by fluorescence rate. Data was recorded in a relative unit per min (RU/min) from control, PO_2_ cycling, Eb, and PO_2_ cycling + H_2_O_2_ (50 µM) treated diaphragm muscle under 40 Torr PO_2_. Fluorescence data was recorded every 5 min (*significantly different from PO_2_ cycling, *P*<0.05; ^#^significantly different from Eb treatment, *P*<0.05; ^†^significantly different from control, *P*<0.05).

Grouped data of mean florescence during 40 Torr PO_2_ are illustrated in Fig. 2A. At the end of 40 Torr PO_2_ periods, ROS levels were elevated from baseline in the control group. However, in the PO_2_ cycling group, ROS levels were kept low compared to control (1.00±0.04 RU *vs.* 1.48±0.05 RU; n = 5 from five animals; *P*<0.05). Furthermore, in the control group, intracellular ROS elevation did not appear within the first 15 min of 40 Torr PO_2_ period. After 20 min, ROS levels were enhanced and these increases lasted until the end of the 30 min period. Fig. 2B displayed the intracellular ROS fluorescence rate (RU/min). In the control group, this rate was close to zero for the first 15 min of 40 Torr PO_2_, followed by three ROS bursts occurring at 20, 25, and 30 min, represented by positive values shown in Fig. 2B (in RU/min). The first ROS burst was relatively smaller (∼50% less) compared to the other two larger bursts. However, in PO_2_ cycling-treated diaphragm muscles, no ROS burst occurred at 40 Torr PO_2_. It is important to note that although Fig. 2B looks similar to Fig. 2A, they refer to two separate measurements: fluorescence in Fig. 2A and fluorescence rate in Fig. 2B. In other words, Fig. 2B shows the mathematical slope of the fluorescence increase/decrease, indicating how fast the signal changes while Fig. 2A shows the level or the intensity of fluorescence.

**Figure 3:**
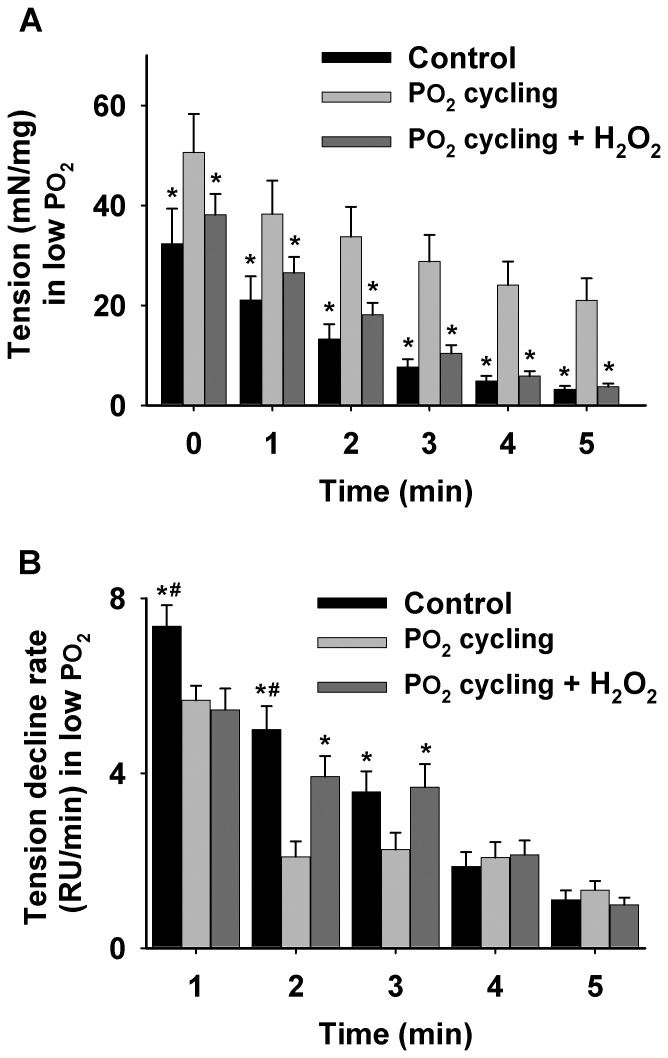
Muscle tension and tension decline rate data under 40 Torr PO_2_. A: absolute tension (mN/mg) was recorded for 5 min from control, PO_2_ cycling, and PO_2_ cycling + H_2_O_2_ group (*significantly different from PO_2_ cycling, *P*<0.05). B: data showing the tension decline rate (RU/min) from control, PO_2_ cycling, and PO_2_ cycling + H_2_O_2_ (50 µM) muscles during a 5-min contractile period under 40 Torr PO_2_ (*significantly different from PO_2_ cycling, *P*<0.05; ^#^significantly different from PO_2_ cycling + H_2_O_2_, *P*<0.05).

**Figure 4:**
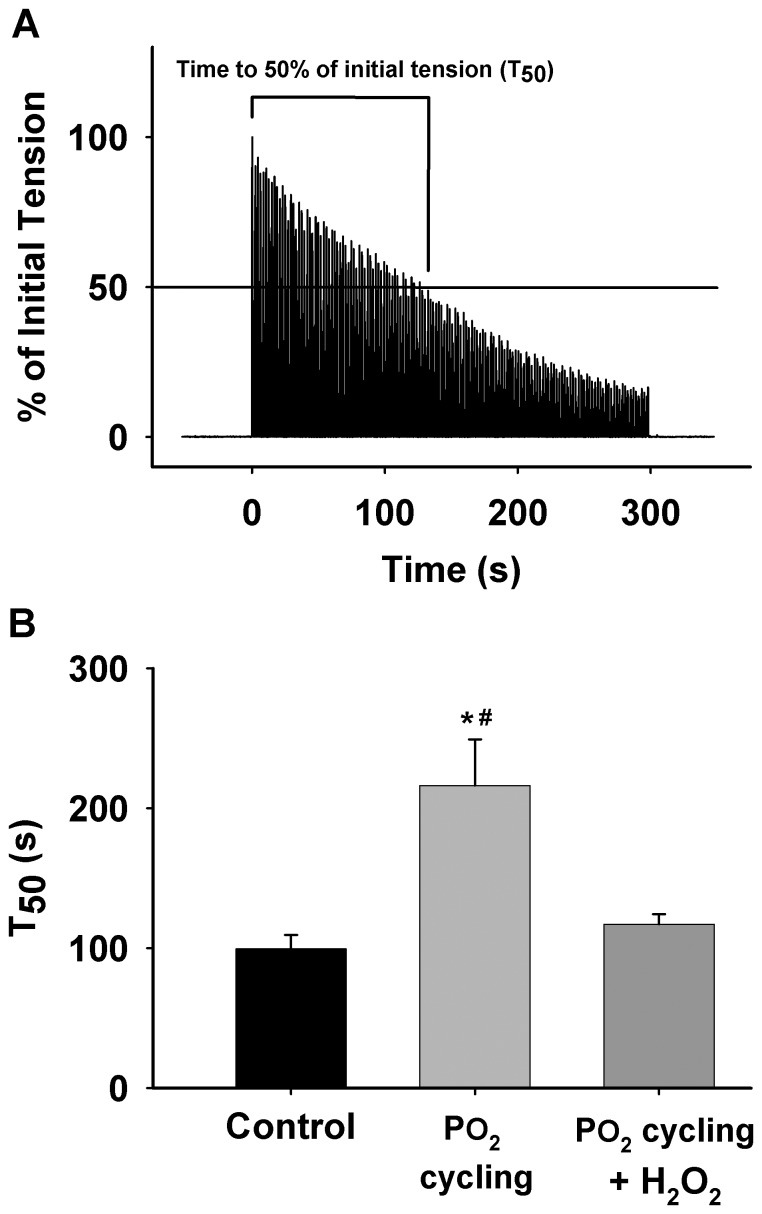
Time to reach 50% (T_50_) of the initial tension in contracting diaphragm muscle under 40 Torr PO_2_. A: a typical chart record illustrating the duration of T_50_ in a contracting diaphragm muscle during a 5-min contraction period. B: summarized T_50_ values from control, PO_2_ cycling, and PO_2_ cycling + H_2_O_2_ (50 µM) groups during the contraction (*significantly different from control, *P*<0.05; ^#^significantly different from PO_2_ cycling + H_2_O_2_, *P*<0.05).

Muscle absolute tension data during 40 Torr PO_2_ are shown in Fig. 3. In the control group, the tension (in mN/mg muscle dry weight) at 0 min and the tension at each subsequent time point thereafter (1–5 min), was significantly lower than the PO_2_ cycling group (n = 5 from five animals, *P*<0.05), suggesting that PO_2_ cycling ameliorated skeletal muscle resistance to fatigue during the 40 Torr PO_2_ period (Fig. 3A). The tension decline rate in PO_2_ cycling treated muscles markedly slowed down in the first 3 min compared to control (n = 5 from five animals for each group, *P*<0.05, Fig. 3B). However, after 3 min the decline rate of all groups was similar, indicating that PO_2_ cycling had no effect on the force decline rate for later fatigue development. Furthermore, the time to 50% of the initial tension (T_50_) from PO_2_ cycling diaphragm muscle was significantly prolonged when compared to control diaphragm (in seconds, 216.2±33.0 *vs.* 99.5±10.0; n = 5 from five animals, *P*<0.05, Fig. 4). However, this difference disappeared in the presence of H_2_O_2_ in the PO_2_ cycling + H_2_O_2_ group (in seconds, 117.1±7.2 vs. 99.5±10.0; n = 5 from five animals, Fig. 4). Maximal diaphragm force was always measured prior to low PO_2_ exposure. The corresponding muscle absolute tension values were reported in Table 1.

**Table 1:** % of maximal force prior to low PO_2_ exposure.

	Control (n = 5)	PO_2_ cycling (n = 5)	PO_2_ cycling + H_2_O_2_ (n = 5)
	Force (mN/mg)	Force (mN/mg)	Force (mN/mg)
	23.7	20.8	37.4
	31.1	45.6	41.0
	61.0	59.6	35.2
	29.8	71.5	51.6
	31.1	41.5	42.9
Average ± SE	35.4±6.56	47.8±8.58	41.6±2.84

We observed that ebselen completely quenched the 40 Torr PO_2_-induced ROS signal in the control (Fig. 1E and F, Fig. 2; n = 4), which demonstrated a similar effect to the PO_2_ cycling treatment group (Fig. 1C and D, Fig. 2, n = 5). The addition of a small amount of H_2_O_2_ (50 µM) entirely abolished the PO_2_ cycling-induced ROS inhibition effect in the confocal experiments (Fig. 1G and H). Grouped data are shown in Fig. 2A. Following 15 min from the onset of 40 Torr PO_2_ period, fluorescence was significantly higher in PO_2_ cycling + H_2_O_2_ group than the control, PO_2_ cycling, and ebselen groups. However, after 30 min of 40 Torr PO_2_ period, the control group showed higher fluorescence than the PO_2_ cycling + H_2_O_2_ and ebselen groups, respectively (n = 5, *P*<0.05). In Fig. 2B, at 15 min during 40 Torr PO_2_ periods, there was a larger fluorescent burst in the PO_2_ cycling + H_2_O_2_ treatment group compared to the other groups. A large fluorescent burst in the control group occurred ∼10 min later than in the PO_2_ cycling + H_2_O_2_, while there were no bursts in ebselen treatment groups, respectively (n = 5, *P*<0.05).

**Figure 5:**
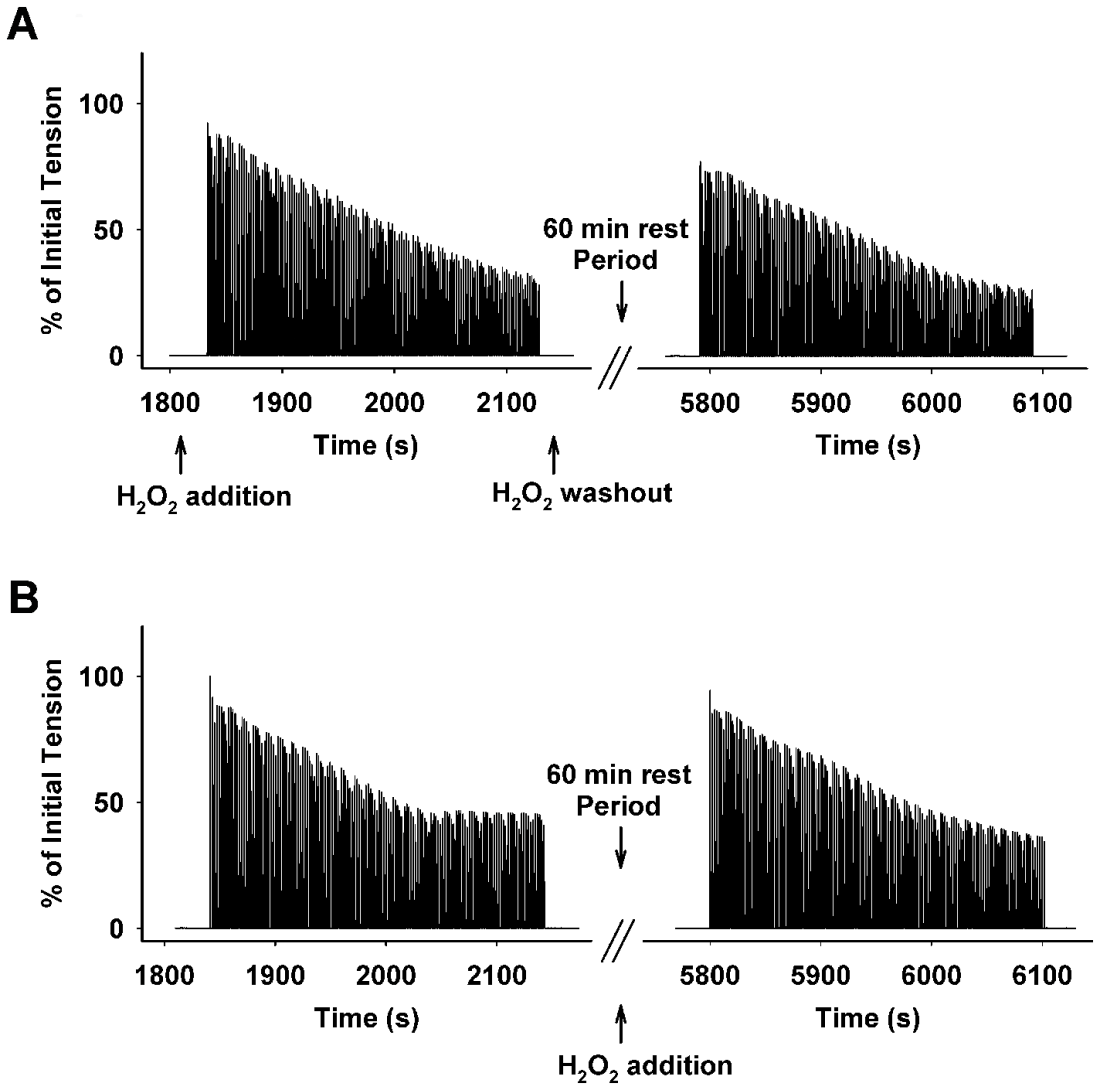
Representative contraction curves showing the effect of H_2_O_2_ (50 µM) on the muscle contraction during high PO_2_ in a blocked order. A: H_2_O_2_ was added 15 min prior to the first 5-min contractile period followed by a H_2_O_2_ washout and 60 min rest period before the second 5- min contractile period in the absence of H_2_O_2_. B: The first 5-min contraction in the absence of H_2_O_2_ was followed by the second 5-min contractile period in the presence of H_2_O_2_.

**Figure 6:**
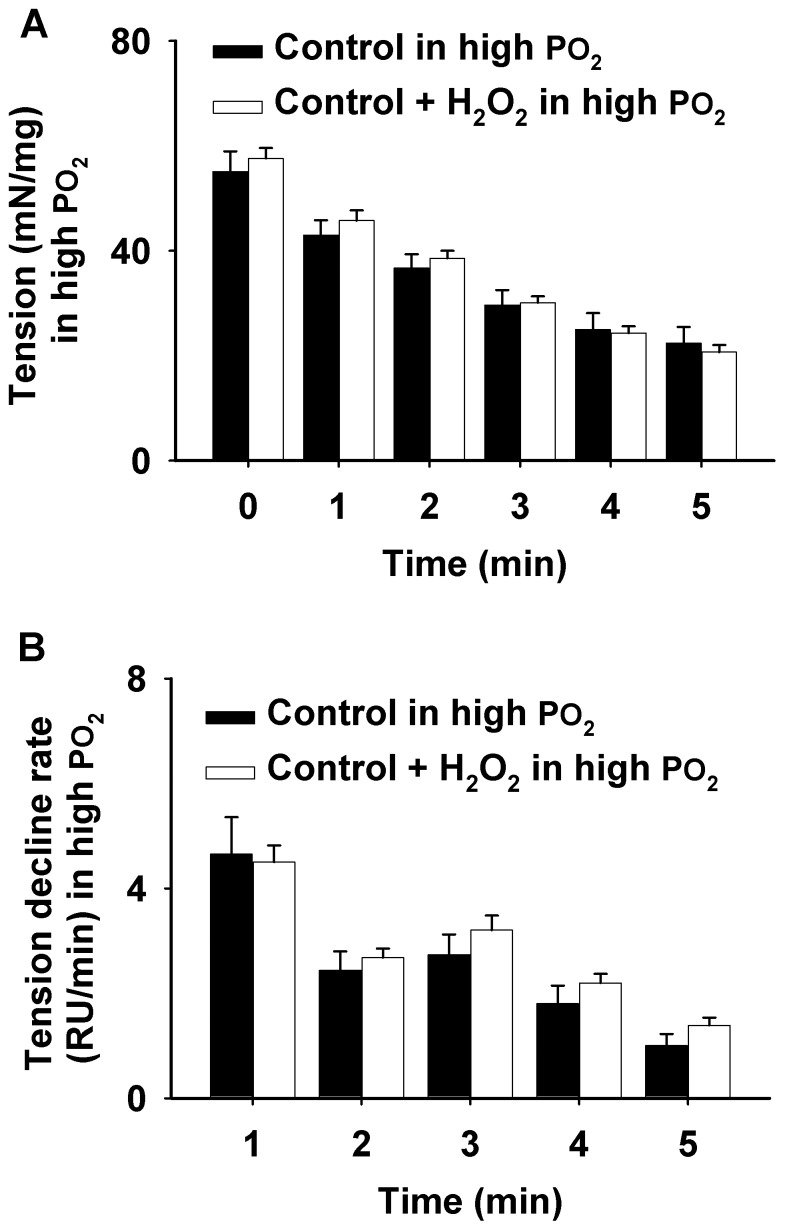
Grouped data showing the effect of H_2_O_2_ (50 µM) on the muscle contraction during high PO_2_ in a blocked order. A: Tension development (mN/mg) at 1–5 min during the 5-min contraction period in the presence vs. absence of H_2_O_2_. B: Tension decline rate (RU/min) during the 5- min contraction period at 1–5 min in the presence vs. absence of H_2_O_2_.

Furthermore, under high PO_2_ conditions (550 Torr), we investigated the effect of a small amount of H_2_O_2_ (50 µM) on muscle contraction as shown in Fig. 5 (representative curves) and Fig. 6 (grouped data). The tension development (mN/mg) and the tension decline rate (RU/min) at 1–5 min during the 5-min contraction were recorded in the presence or absence of H_2_O_2_. Both figures clearly illustrate that H_2_O_2_ had no marked effect on muscle function at a level of 50 µM (n = 6).

**Figure 7:**
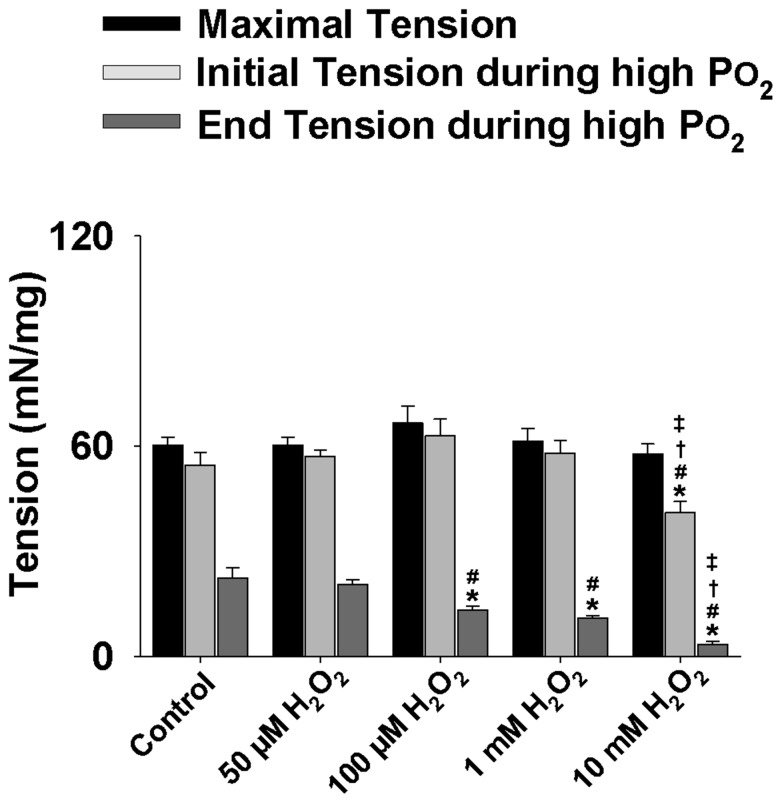
Grouped data showing the effect of varying H_2_O_2_ dosage on muscle tension development during high PO_2_. Data showing the muscle tension development (mN/mg) during the H_2_O_2_ dosage treatments in mouse diaphragm strips [0 µM (control), 50 µM, 100 µM, 1 mM and 10 mM] in high PO_2_ (550 Torr). *Significantly different from control (*P*<0.05). ^#^Significantly different from 50 µM H_2_O_2_ (*P*<0.05). ^†^Significantly different from 100 µM H_2_O_2_ (*P*<0.05). ^‡^Significantly different from 1 mM H_2_O_2_ (*P*<0.05).

Moreover, H_2_O_2_ dosage experiments were performed in a range from 0 µM to 10 mM under high PO_2_ conditions (550 Torr) as shown in Fig. 7. Muscle tension development was recorded for the maximal contraction during the baseline period and the initial and end contractions during the 5-min contraction period. There was no significant difference between the control group (0 µM) and the 50 µM group. However, the 100 µM and 1 mM groups (n = 9 for both groups) did show a significant decrease in muscle function during the end contraction when compared to both the control and 50 µM groups (*P*<0.05). The greatest declines in muscle tension were observed in the 10 mM group (n = 8) as both the initial and end contractions showed a marked decrease in muscle tension development in comparison to all other dosage groups (*P*<0.05).

## Discussion

The current study provides evidence that the PO_2_ cycling preconditioning procedure we used reduces intracellular ROS levels in respiratory skeletal muscle during prolonged low PO_2_. The absolute skeletal muscle tension and T_50_ were both greater in the PO_2_ cycling group than the control group, but the addition of a small amount of ROS (H_2_O_2_) reduced these values to control levels. However, this amount of ROS was so marginal that it exerted no significant effect on muscle function during hyperoxia. Collectively, these data indicate that the protection of PO_2_ cycling on the diaphragm is related to the reduced levels of intracellular ROS signaling molecules.

Dihydrofluorescein (Hfluor) is a highly sensitive intracellular probe commonly used for fluorescent detection of ROS. Fluorescein (Fluor) formation results when Hfluor reacts with ROS [5]. Previous research has shown that Hfluor is much less sensitive to nitric oxide (NO) compared to its analog dichlorfluorescein (DCFH) and also shows a higher resistance to photobleaching than DCFH [5], [18], [19]. Since it is superior for detecting ROS (particularly H_2_O_2_), it was used in our experiments. Our results showed that there was no marked ROS formation in the muscle during the first 15 min of a 40 Torr PO_2_ period. In the control group (Fig. 2A), ROS levels were significantly increased after 20 min from the initiation of 40 Torr PO_2_, which may suggest that during this timeframe antioxidant defenses were overwhelmed in diaphragm muscle not treated with PO_2_ cycling. Moreover, the completely abolished ROS signals in the antioxidant (ebselen) treated control group (Fig. 1 and 2), confirms the existence of ROS, which seems to be quenched by PO_2_ cycling treatment in our experiments (Fig. 1 and 2). Interestingly, after PO_2_ cycling treatment, extracellular addition of a small amount of ROS (50 µM H_2_O_2_), which has no effect on normal muscle function (Fig. 5 and 6), completely negated the PO_2_ cycling effect. These observations suggest to us that PO_2_ cycling may be involved in the initiation of intracellular antioxidant signaling pathways.

It should be noted that the experimental conditions used to detect ROS are different from those used to evaluate skeletal muscle function in our settings. ROS detection was performed in unstimulated muscle, while function was assessed in contracting muscle, for the following reasons: 1) Due to large motion artifact, it is extremely difficult to perform muscle function experiments under confocal microscopy; 2) The muscle function experiment is focused on measurement of maximal force and time to fatigue. However, the confocal experiment is primarily designed to determine intracellular ROS levels in the muscle.

Evidence has shown that PO_2_ cycling triggers the expression of superoxide dismutase (SOD), an endogenous antioxidant, which may further contribute to reduced ROS levels [7], [10], [20]. This is highly consistent with our observations of reduced ROS level in PO_2_ cycling treated skeletal muscle. Similar to heart studies which show that both IPC and PO_2_ cycling are mediated by ROS [7], intracellular ROS levels were also critical for PO_2_ cycling efficacy in diaphragm muscle. There is a potential concern that PO_2_ cycling could have altered mitochondrial function and integrity. However, our muscle function data (Fig. 3 and 4) suggest that fatigue resistance in PO_2_ cycling treated mouse diaphragm muscle was substantially greater than that of control muscle. Thus, it is likely that mitochondrial activity was not negatively altered by PO_2_ cycling treatment. Although in the current study design we are unable to determine whether PO_2_ cycling treatment causes decreased ROS production or increased antioxidant scavenging, it is likely that there is a specific redox mechanism that suppresses ROS generation during prolonged respiratory muscle exposure to the 40 Torr PO_2_. The detailed mechanism associated with PO_2_ cycling protection, however, is still unclear and requires further study. In addition, based on a similar previous study of PO_2_ levels in myocytes, 40–550 Torr PO_2_ was an effective setting to initiate intramuscular redox changes in skeletal muscle [21], [22].

Interestingly, we noticed that at 15 min during the 40 Torr PO_2_ period, ROS levels were significantly higher in the PO_2_ cycling + H_2_O_2_ group compared to the other groups; yet, at 30 min, the control group showed a higher ROS level than the PO_2_ cycling + H_2_O_2_ group (Fig. 2A). This suggests that the two treatment plans stimulate a time-dependent intracellular ROS formation mechanism. In addition, we evaluated the ROS generation rate, defined as a ROS burst and represented by the fluorescence rate, as shown in Fig. 2B. H_2_O_2_ addition after PO_2_ cycling treatment induced the first ROS burst at 15 min under the 40 Torr PO_2_ conditions, which occurred ∼10 min earlier than a large ROS burst in the control group. Collectively, these observations suggest a faster diffusion of extracellular ROS (H_2_O_2_) into the intramuscular compartment, compared to the intracellular ROS generation in the control muscle. However at a later time (after 25 min during low PO_2_), the control muscle showed a higher ROS formation rate than the PO_2_ cycling + H_2_O_2_ group. This could be due to leakage of H_2_O_2_ into the perfusate. Nevertheless, these data further demonstrate a potential antioxidant-like effect exerted by PO_2_ cycling, and this effect can be disturbed by a small addition of ROS, which was not sufficient to influence muscle contractility (Fig. 5 and 6).

Moreover, it is suggested that PO_2_ cycling mitigates fatigue within the diagram during hypoxia. Although the exact mechanism by which this occurs is unclear, it is likely that ROS play a significant role. Accordingly, our data (Fig. 1 and 2) suggest that low levels of ROS negate the benefits of PO_2_ cycling and may be involved in other signaling events, including antioxidant cascades. Figures 5 and 6 illustrate the relationship between ROS level and muscular force generation. These results are supported by previous studies in which it was concluded that low levels of H_2_O_2_ may work more towards signaling pathways since they do not directly impact force generation in the muscle [23], [24]. Further research into the effect of both ROS and PO_2_ cycling on diaphragm force generation and related muscular mechanisms may lead to potential therapies to mitigate muscle fatigue during hypoxia.

The H_2_O_2_ dosage experiments (Fig. 7) on the diaphragm function suggest that high levels of ROS (H_2_O_2_) such as at 1 mM or 10 mM levels markedly reduce muscle function; however, lower levels of ROS, such as 50 µM, do not compromise muscle function as compared to control. In addition, our confocal experiments have clearly shown that this low level of ROS (50 µM) minimized the PO_2_ cycling effect confirming that rather than damaging muscle directly, low levels of ROS may be a potential mediator for the signaling events involved in PO_2_ cycling preconditioning.

There are a number of intracellular sources of ROS in skeletal muscle, including the mitochondria, xanthine oxidase (XO), peroxisomes, and NADPH oxidase [25]. For example, under respiratory stress, such as ischemia or low PO_2_/hypoxia, xanthine dehydrogenase converts to XO, which is subsequently released into circulation and produces ROS [22], [25]. In skeletal muscle myocytes, NADPH oxidase is another likely candidate for ROS formation during hypoxic stress during injuries [23]. Additionally, the mitochondria produce low levels of superoxide anion under normal conditions [26], but in the lack of oxygen, the mitochondria experience excessive oxidant production [5], [26], [27]. Accordingly in such a condition, the mitochondria function as a source of increased level of superoxide, one of the common precursors to most ROS. This increase in intracellular ROS could potentially overwhelm natural antioxidant defense systems, leading to impaired muscle function [2], [28]–[30]. Our data (Fig. 2B) showed that three ROS oxidative bursts (represented by positive rate values) occurred after 15 min of the 40 Torr period in the control muscle, which may indicate that intracellular antioxidant defenses have a 15 min effective period until they are eventually negated by subsequent ROS formation. This timeframe is within the regular activation time range of mitochondrial antioxidant enzymes [30], [31]. Therefore, both XO and mitochondria are possible sources of ROS in this study.

Moreover, our findings have shown considerable evidence regarding the protective effects of PO_2_ cycling training on skeletal muscle function (Fig. 3A). During the middle of the 40 Torr PO_2_ period, the initial tension of the PO_2_ cycling treated muscle strips was higher than that of control, and this trend kept increasing to ∼4 fold greater than control from 1- to 5-min suggesting that PO_2_ cycling progressively alleviated muscle fatigue. However, after H_2_O_2_ was loaded into the muscle at a relatively low dosage (Fig. 5 and 6), the protection from PO_2_ cycling was diminished. This supports that the ROS signaling molecules may possibly play a negative role in the PO_2_ cycling mechanism. PO_2_ cycling markedly reduced the tension decline rate at both 1-, 2- and 3-min from the initial contraction compared to control. These rate differences were minimized at 4- and 5-min, respectively. The addition of H_2_O_2_ also significantly interrupted the PO_2_ cycling effect on the tension decline rate at 2- and 3-min in the middle of the 40 Torr PO_2_ period (Fig. 3B), confirming that ROS signaling molecules adversely affect PO_2_ cycling pathways. A possible correlation among PO_2_ cycling, muscle fatigue and ROS implies that PO_2_ cycling is able to boost muscle contractility during fatigue, which is consistent with Clanton's and Reid's results [1], [32]. Specifically, PO_2_ cycling could result in a gradual increase in the production of endogenous antioxidant enzymes, allocating the additional antioxidants to serve as a “reservoir” that can be promptly accessed in response to spontaneous exposure to stressful conditions. This idea is consistent with previous research suggesting that PO_2_ cycling significantly increases the expression level of intramuscular antioxidants such as SOD [7].

Previous research has shown that the intramuscular PO_2_ during strenuous exercise is ∼4 Torr, while ∼30 Torr conditions are seen in resting muscle [33]. In the current research, we created a relatively lower PO_2_ condition by equilibrating 40 Torr PO_2_ and a hyperoxic condition with 550 Torr based on previous studies in skeletal muscle [5], [15], [21], [34]. However, the intracellular PO_2_ was difficult to determine under our experimental set-up, particularly in a contracting muscle with marked motion. During exhaustive exercise, skeletal muscle conditions fluctuate between higher PO_2_ and lower PO_2_, which frequently occur especially during high intensity interval trainings. This cycling in intracellular oxygen exerts an intrinsic preconditioning effect, similar to the PO_2_ cycling protocol implemented in our studies. Additionally, it has been shown that increased levels of catalase and SOD are expressed in skeletal muscle during exercise training, resulting in a reduction of ROS level and oxidative stress [35]–[38]. Similar to exercise training, PO_2_ cycling therapy may be an alternative method for increasing muscular endurance. Moreover, it is worth noting that a small amount of ROS (50 µM H_2_O_2_) has no effect on normal muscle function (Fig. 5 and 6). However, this dosage completely abolished the PO_2_ cycling protection in low PO_2_ conditions (Fig. 3). The exact mechanism of this response is still unknown, which will be an area of future studies.

Some limitations appear in our study. First, we are unable to determine whether 40 Torr in the solution can cause intramuscular hypoxia. Second, it is possible that our PO_2_ cycling protocol, by itself, can directly affect intracellular ROS and muscle fatigue without additional mediators. Third, it is difficult to measure the exact intracellular PO_2_ level in a functioning diaphragm. This is mainly because of a marked O_2_ diffusion gradient across the multiple layers of diaphragm tissue. Although previous research has shown that in a similar condition to 40 Torr PO_2_, intracellular levels of NADH in the diaphragm significantly increase [5], it is not clear whether 40 Torr PO_2_ in the superfusate can cause the intracellular compartments hypoxic. Lastly, since O_2_ cannot transport across the different layers equally in the whole muscle, it is more likely that a hypoxic condition may occur in the core of the muscle than the peripheral region [39]. In addition, intracellular PO_2_ in skeletal muscle is ∼10 Torr at rest, but it quickly drops to 3–5 Torr during intense exercise [40]. It is likely that the transition between 550 Torr to 40 Torr triggers mismatches of oxygen supply to the diaphragm, which may be sufficient to induce a transient ROS formation as described in our earlier research [5]. Our study demonstrated that intracellular ROS is elevated in single myofibers during a similar PO_2_ condition [21]. Interestingly, this level of oxygen (3–5 Torr) is regarded as normal for exercising human muscles [33]. Precisely controlling the intramuscular O_2_ condition within the whole muscle preparation is highly challenging and therefore should be the focus of future research.

### Perspectives and Significance

This study demonstrates that PO_2_ cycling mediates beneficial responses through reducing intracellular ROS levels in respiratory muscle. PO_2_ cycling is a drug-free treatment that possibly stimulates the diaphragm to activate its own antioxidant defense systems to resist fatigue development. This may be an effective method for enhancing muscular endurance. In addition, the current *in vitro* study provides a redox perspective into mouse respiratory muscle under optimal preconditions.

## Supporting Information

Dataset S1 **ROS fluorescence (** **figures 1** **and** **2** **).**
(XLSX)

Dataset S2 **Muscle tension and tension decline rate (** **figure 3** **).**
(XLSX)

Dataset S3 **Time to reach 50% (T_50_) of the initial tension (** **figure 4** **).**
(XLSX)

Dataset S4 **Representative H_2_O_2_ (50 µM) contraction curves (** **figure 5** **).**
(XLSX)

Dataset S5 **Grouped H_2_O_2_ (50 µM) data (** **figure 6** **).**
(XLSX)

Dataset S6 **Grouped data of varying H_2_O_2_ dosage (** **figure 7** **).**
(XLSX)
